# Supplementary material for: Early Weaning Inhibits Intestinal Stem Cell Expansion to Disrupt the Intestinal Integrity of Duroc Piglets via Regulating the Keap1/Nrf2 Signaling
Source: Antioxidants (Basel). 2024 Sep 30;13(10):1188. doi: 10.3390/antiox13101188 (PMC11505184; doi:10.3390/antiox13101188)
Supplement: Supplementary file 1 [file antioxidants-13-01188-s001.zip › antioxidants-3188779-supplementary.pdf]

Supplementary Materials

Table S1. Antibody information

Antibody	gene accession	Source	Product Number
zonula occludens-1 (ZO-1)	NC-000073	#339100	Thermo Fisher
Claudin 1	NC_000003	#374900	Thermo Fisher
Cleaved Caspase3 (C-Caspase3)	NC-000074	#9664	Cell Signaling Technology
Olfactomedin4 (Olfm4)	NC-052532	14369	Cell Signaling Technology
Keratin20 (KRT20)	NC-010454	13063	Cell Signaling Technology
Villin	NC-000067	#sc-58897	Santa Cruz
Occludin	NC-000079	#502601	Zen BioScience
SRY-box transcription factor 9 (SOX9)	NC-000017	#380995	Zen BioScience
Proliferating Cell Nuclear Antigen (PCNA)	NC-000020	#200947-6B12	Zen BioScience
Superoxide Dismutase 1 (SOD1)	NC-000021	#R25829	Zen BioScience
Keap1	NC-000019	#R26935	Zen BioScience
p-Nrf2	NC-000002	#381559	Zen BioScience
Nrf2	NC-000068	#380773	Zen BioScience
Heme oxygenase-1 (HO-1)	NC-000022	#380753	Zen BioScience
β-actin	NC-000071	#600149	Zen BioScience
secondary antibodies anti-rabbit IgG	/	#511203	Zen BioScience
anti-mouse IgG	/	#511103	Zen BioScience

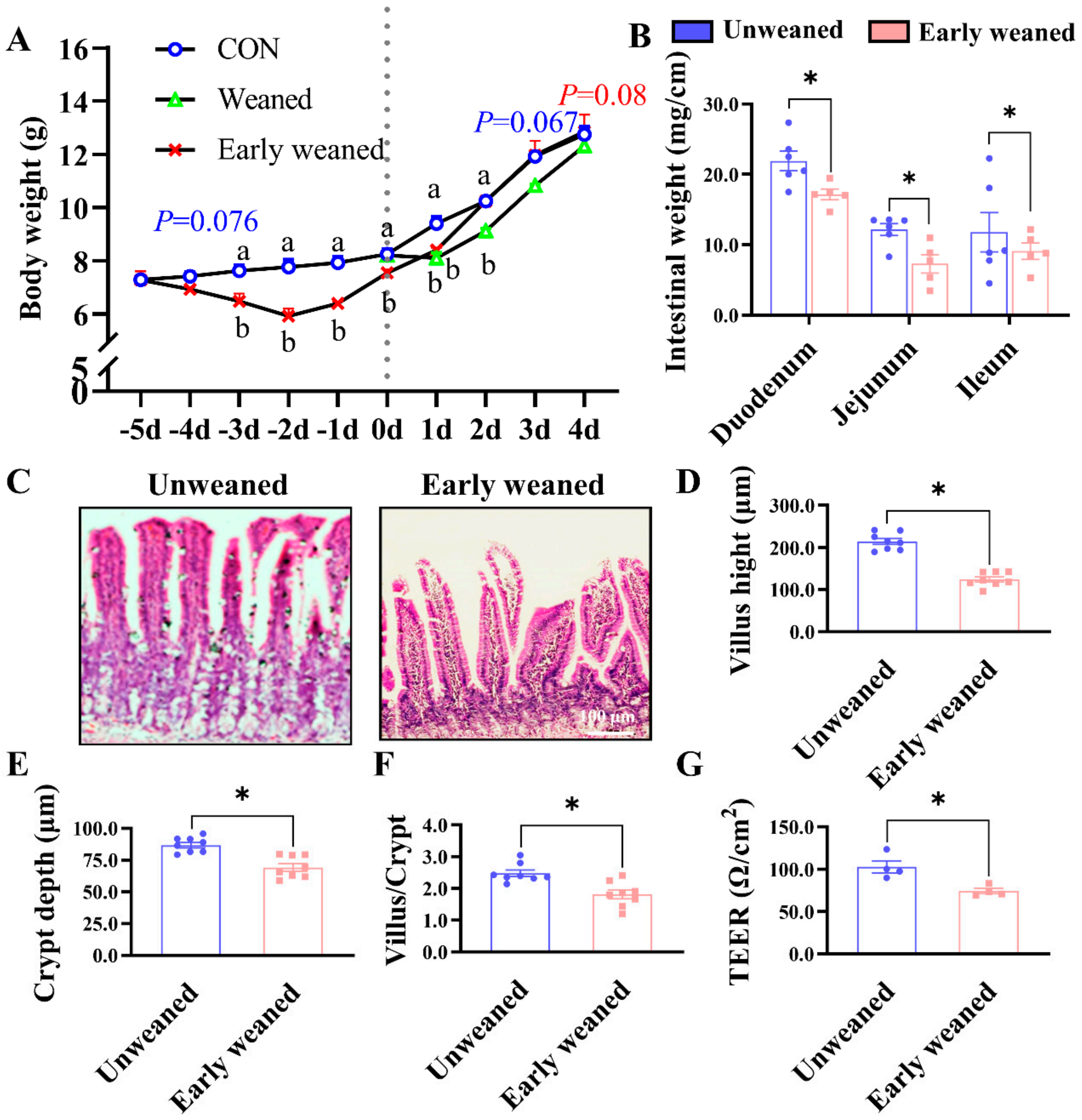

Figure S1. Early weaning destroys the intestinal structure of mice. (A) Early weaned and weaned reduced the body weight of mice. (B) Intestinal weight per centimeter (n = 6 mice). (C) Representative images of H&E staining in the jejunum of piglets are shown. (D-F) The results of the statistical analysis of villus height (D), crypt depth (E), and the ratio of the villus to crypt (F). (G) The TEER of jejunum. The results are expressed as the mean ± SEM (n = 4 mice). *$P < 0.05$.

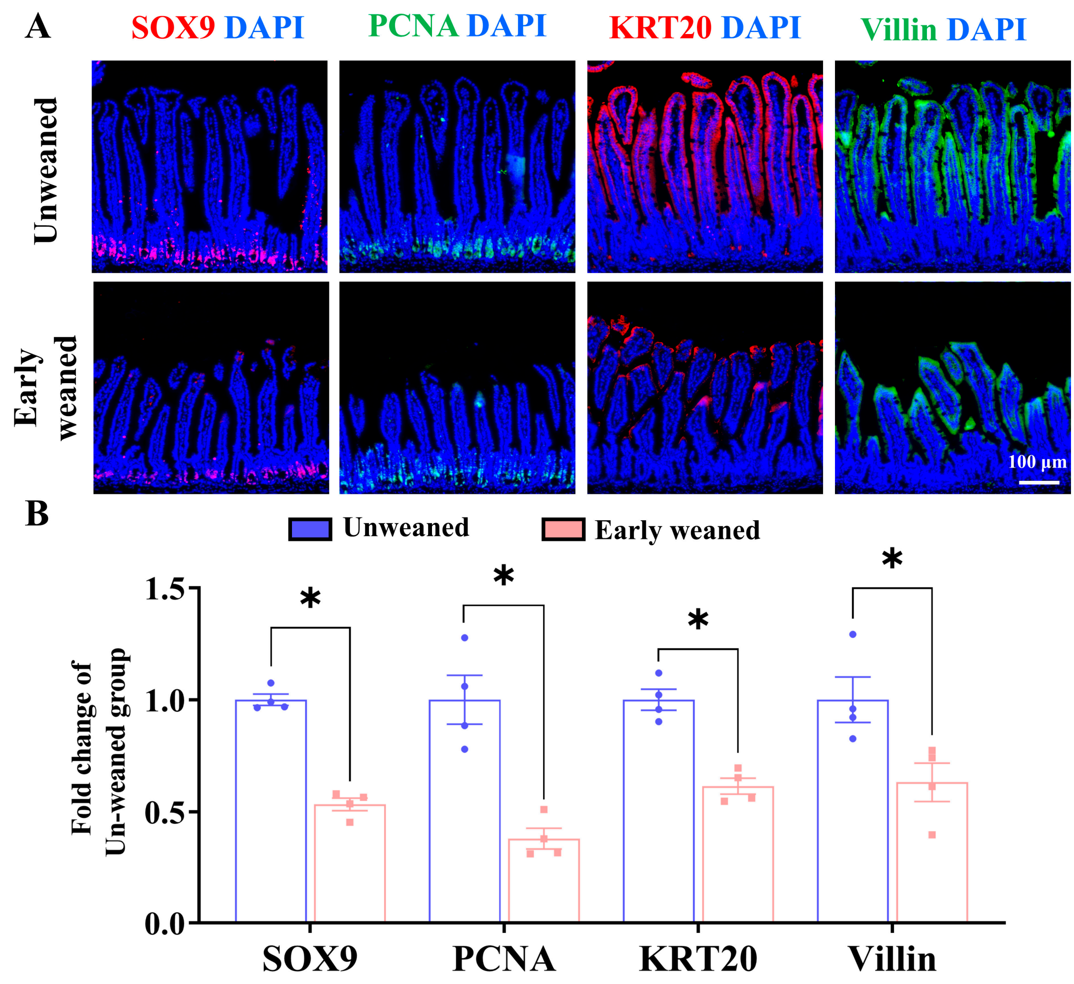

Figure S2. Early weaning decreases the levels of various marker proteins in the jejunum of mice. (A-B) The expression level of related proteins such as SOX9, PCNA, KRT20, and Villin in the jejunum was detected by IHC. The results are expressed as the mean ± SEM (n = 4 mice). Each respective Un-weaned group was set at 1. *$P < 0.05$.

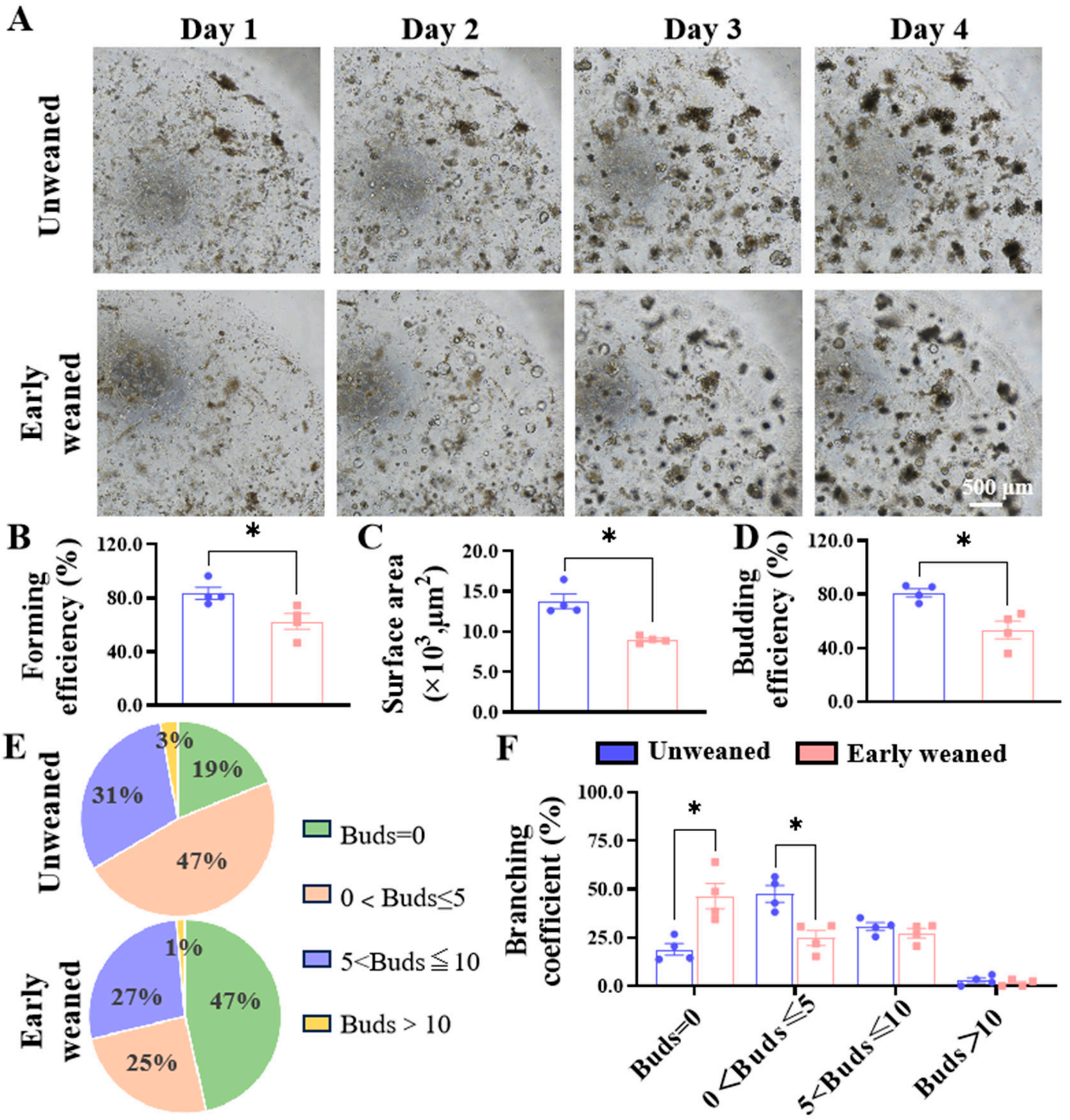

Figure S3. Early weaning inhibits intestinal stem cell proliferation in mice. We performed early weaning of 16-day-old male mice and isolated the jejunal crypts in each group of mice after weaning 3 Days. (A) Representative images of intestinal organoids cultured from crypt cells in each group of mice on days 1, 2, 3, and 4 are shown. (B-F) The organoid forming efficiency was measured on Day 2 (B); Surface area (C), budding efficiency (D), bud number (E), and branching coefficient of organoids were measured on Day 4 (F). The results are expressed as the mean ± SEM (n = 4 mice). *$P < 0.05$.

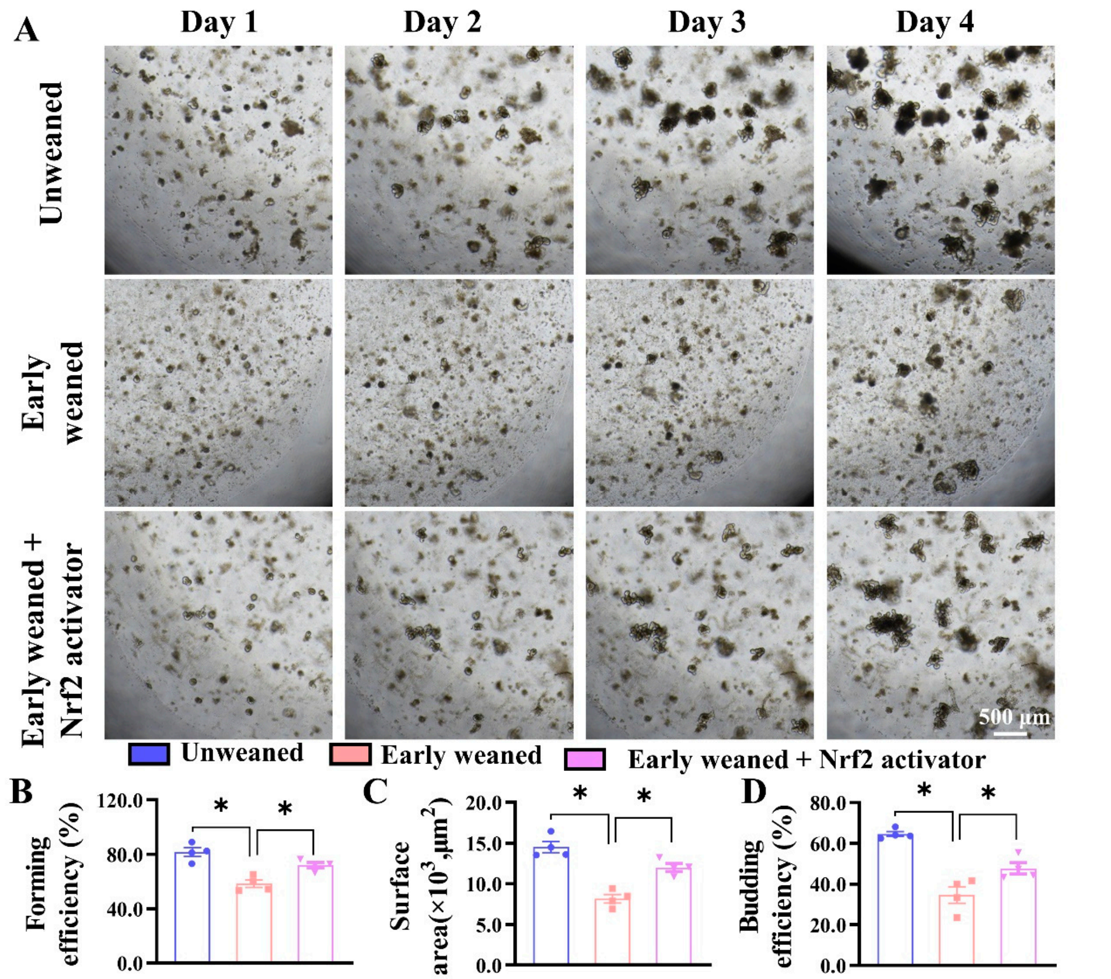

Figure S4. Nrf2 mediates weaning stress-induced intestinal stem cell injury of mice.

We performed early weaning of 16-day-old male mice and isolated the jejunal crypts in each group of mice after weaning 3 Days. *Ex vitro* treatment of intestinal organoids in the early weaning group with Nrf2 activator to alleviate weaning stress damage in intestinal stem cells. (A) Representative images of intestinal organoids cultured from crypt cells in each group of piglets on days 1, 2, 3, and 4 are shown. (B-D) The organoid forming efficiency was measured on Day 2 (B), Surface area (C), and budding efficiency (D). The results are expressed as the mean ± SEM (n = 4 mice). *$P < 0.05$.
